# Landscape of immune checkpoint inhibitor-related adverse events in Chinese population

**DOI:** 10.1038/s41598-020-72649-5

**Published:** 2020-09-23

**Authors:** Li Li, Gang Li, Bin Rao, An-Hui Dong, Wei Liang, Jin-Xian Zhu, Mu-Ping Qin, Wen-Wen Huang, Jie-Ming Lu, Zi-Fang Li, Yao-Zhong Wu

**Affiliations:** 1 Department of Radiation Oncology, Wuzhou Red Cross Hospital, 3-1 Xinxing First Road, Wuzhou, 543001 People’s Republic of China; 2 Department of Medical Oncology, Wuzhou Red Cross Hospital, 3-1 Xinxing First Road, Wuzhou, 543001 People’s Republic of China; 3 Department of Breast Surgery, Wuzhou Red Cross Hospital, 3-1 Xinxing First Road, Wuzhou, 543001 People’s Republic of China

**Keywords:** Cancer therapy, Tumour immunology

## Abstract

This study aimed to describe the landscape of Immune checkpoint inhibitors (ICIs)-related adverse events (AEs) in a predominantly Chinese cohort. We searched electronic datasets including PubMed, Web of Science and Embase to identify and recruit relevant trials up to September 2, 2019. Clinical trials focusing on ICIs in Chinese patients or a predominantly Chinese population were included. Incidences of treatment-related AEs (TRAEs) and immune-related AEs (irAEs) were pooled and compared. In total, we recruited 13 trials consisting of 1063 patients, with 922 (86.7%) receiving ICI monotherapy and 141 (13.3%) receiving combination of ICI with chemotherapy or anti-angiogenesis. The pooled incidence of any grade TRAEs, grade 1–2, grade 3–5 TRAEs, any grade irAEs, grade 1–2 irAEs and grade 3–5 irAEs in all 1063 patients were 84.1%, 63.3%, 20.9%, 43.3%, 40.0% and 3.0%, respectively. Moreover, 4.3% (44/1018) of patients experienced treatment discontinuation and only 8 (0.8%) patients experienced treatment-related death. Compared to ICI monotherapy, combination significantly increased grade 3–5 TRAEs (46.1% vs. 17.0%, *P* < 0.001) and grade 3–5 irAEs (7.1% vs. 2.0%, *P* = 0.015). By comparing the toxicity profiles between different ICIs, we found some drug-specific AEs such as reactive capillary haemangiomas for camrelizumab (58.6%), hyperglycemia for toripalimab (55.6%) and pyrexia for tislelizumab (54.3%). Additionally, nivolumab has the lowest incidence of any grade (64.1%) and grade 3–5 (11.8%) TRAEs. ICI-related AEs were generally mild and tolerable for a predominantly Chinese cohort. However, we should pay attention to the combination of ICI with chemotherapy as it could increase grade 3–5 TRAEs and irAEs.

## Introduction

Cancer has become as the leading cause of death and the single most important barrier to health life expectancy in every country of the world in the twenty-first century^1^. Also, the increasing cancer incidence and mortality has made cancer the leading cause of death and a major public health problem in China^2,3^. In recent years, the advances in early detection and treatment as well as the growth and aging of the population has enabled the increasing of cancer survivors in developed countries like the USA^4^. However, the scenario is different in less developed countries like China because the underdeveloped economy and poor medical care constrain the access to routine cancer screening and novel and effective anti-tumor treatments. Therefore, cancer treatment still remains a huge challenge and threat in China.

Over the past 5 years, immunotherapy represented by immune checkpoint inhibitors (ICIs) targeting programmed death 1 (PD-1), programmed death ligand-1 (PD-L1) and cytotoxic T lymphocyte-associated antigen-4 (CTLA-4) has become an important milestone in cancer treatment^5^. Different from conventional cytotoxic drugs, ICI enhances the autoimmune power to kill cancer cells through blocking negative regulators expressed on immune or tumor cells. Currently, ICI (nivolumab, pembrolizumab, atezolizumab, durvalumab and ipilimumab) alone or combined with chemotherapy has become the standard first-line or second-line therapies for various malignancies^6–17^. The durable response to ICI makes cancer a “chronic disease” for a small proportion of patients^18^. Despite the impressive efficacy, ICI-related toxicities (i.e., immune-related adverse events, irAEs) should not be neglected because many like myocarditis and pneumonitis are covert and fatal^19^. Having a knowledge of these fatal irAEs is the key for successful application of ICI in clinical practice.

Although ICI has been applied in clinical practice for a few years, safety data from Chinese cohorts were very rare. Given the heavy tumor burden and strong need for ICI, it is therefore of great importance and clinical significance to describe the safety profile of ICIs in Chinese patients. Here, we report the landscape of irAEs in a predominantly Chinese cohort to provide a preliminary understanding and aid clinicians in improving early recognition and management of irAEs through a comprehensive review of published literatures.

## Materials and methods

### Online datasets and searches

Three investigators (LL, GL and BR) independently searched the online datasets including PubMed, Web of Science and Embase to include relative clinical trials which focused on ICI and were published in English up to September 22, 2019. Conference abstract books were not searched. The searching key words were “Immune Checkpoint Inhibitor OR immunotherapy OR PD-1 OR PD-L1 OR anti-programmed death 1 OR anti-programmed death ligand-1 OR CTLA-4 OR cytotoxic T lymphocyte-associated antigen-4” AND “Cancer OR Tumor OR Carcinoma OR Malignancy OR Neoplasm OR Lymphoma OR Sarcoma”. All studies were restricted to clinical trials.

### Study inclusion

Only clinical trials (Phase I-IV) focusing on ICI (alone or combined with other anti-tumor treatments such as chemotherapy and anti-angiogenesis) in patients with malignant disease were included because safety data from trials may be more accurate and reliable than that from retrospective studies. ICIs approved or not approved by current guidelines were both included for analysis. Conference abstracts, posters or oral presentations of ongoing trials without publication were excluded because they did not provide detailed safety data. At the beginning, we planned to include studies recruiting only Chinese patients. However, the number of studies meeting such criteria is too small, and studies with a predominantly Chinese population were therefore also included. Moreover, studies only reporting treatment-related adverse events (TRAEs, defined as all adverse events which were consider to be associated with immune checkpoint inhibitors, including irAEs and non-irAEs [such as vomiting, leukopenia, dyspnea and so on]) but not irAEs (defined as the toxicities caused by ICIs-induced abnormal autoimmune such as autoimmune colitis, pneumonitis, thyroiditis and so on) were also excluded.

### Data extraction

The study data including disease, treatment and toxicity information were extracted by two authors (LL and A-PQ) separately. For randomized clinical phase II/III trials comparing ICI with other conventional treatments, we only extracted the safety data of ICI arm. If the study stated in the text that AEs with an incidence below 5% or 10% were not reported, the study sample size would not be included in calculation of overall incidence of corresponding AEs. If the study had no such statement, we would treat the corresponding incidence of not reported AEs as 0 and include the study sample size into calculation of overall incidence. The following items would be presented in our study: first author, published journal and year, disease, study period, study phase, region, treatment (drug and dose), sample size, TRAEs and irAEs. All toxicities were graded according to National Cancer Institute Common Terminology Criteria for Adverse Events (CTCAE, version 4.0 or 4.03).

### Study endpoint and statistical method

The major endpoint of current study was TRAEs and irAEs in the whole pooled cohort, ICI monotherapy and ICI combination cohorts. Other assessed outcomes were comparisons of TRAEs and irAEs profiles between ICI monotherapy and ICI combination cohorts, and between different ICIs in monotherapy cohort. AEs were pooled up per ICI and regimen described in percentage. Chi-square test or Fisher exact test was used to perform the comparison. Statistical analysis were performed using Statistical Package for the Social Science, version 20.0 (SPSS, Chicago, IL, USA) and. Two-sided *P* < 0.05 indicated a statistically significant difference.

## Results

### Baseline information of included studies

**Figure 1:**
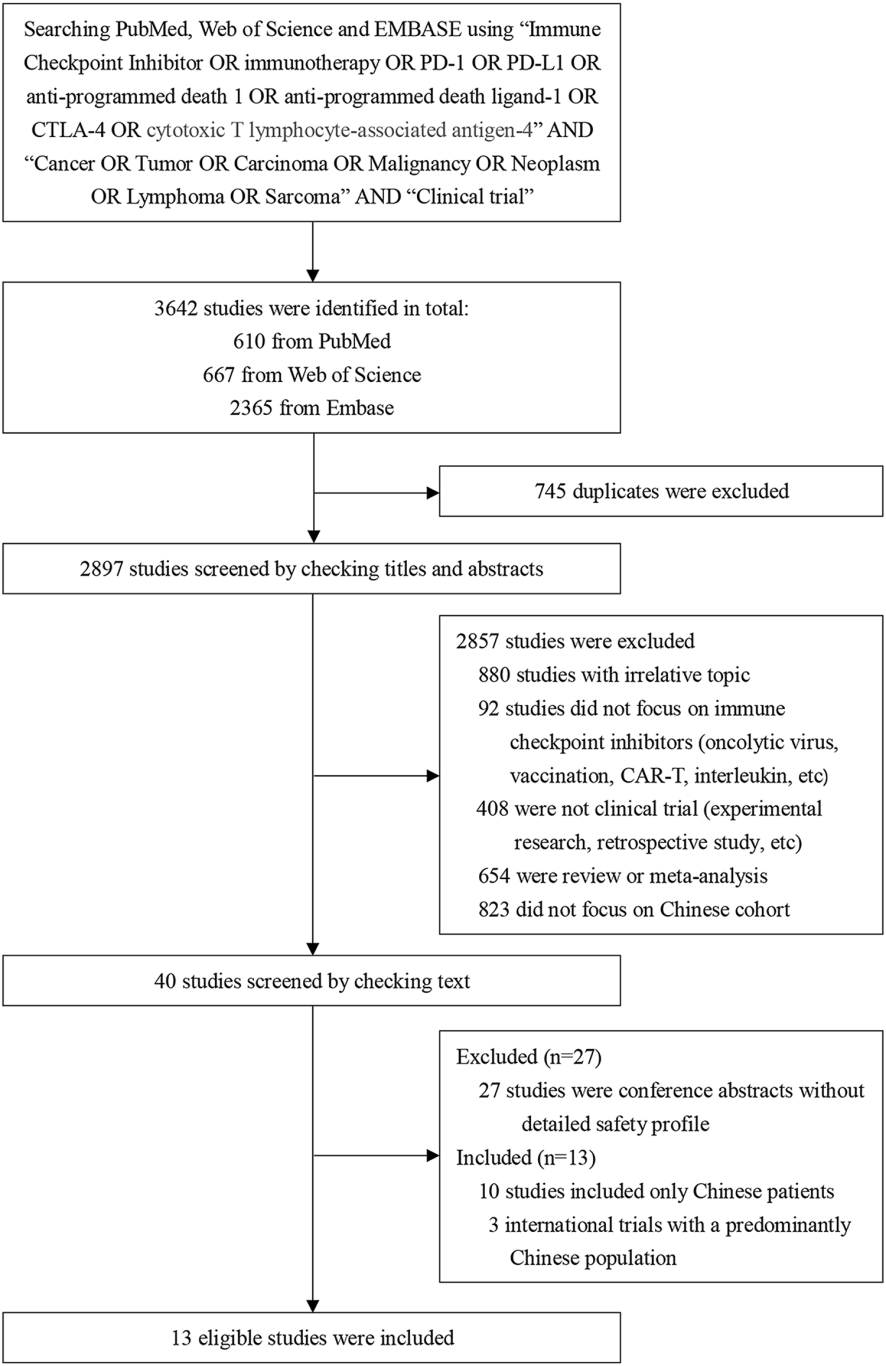
Flow chart of study inclusion.

Up to September 22, 2019, we totally identified 40 potentially eligible studies (Fig. 1). Twenty-seven of them were excluded as they were conference abstracts, leaving 13 eligible studies consisting of 1063 patients for analysis^20–32^. Basic information of these studies were shown in Table 1. Overall, there were six ICIs were assessed including pembrolizumab (n = 27), nivolumab (n = 382), camrelizumab (n = 343), toripalimab (n = 145), tislelizumab (n = 70) and sintilimab (n = 96). Among these studies, there are three international trials^20,23,32^ and only one phase III trial (Checkmate-078, nivolumab)^32^. More than half of them focused on nasopharyngeal carcinoma (NPC, n = 3) or classical Hodgkin lymphoma (cHL, n = 4). Three studies have two arms^21,25,31^ and four studies focuse on the combination of ICI with chemotherapy^21,25,31^ or anti-angiogenesis^26^. Keynote-028 is the only trial that requires positive PD-L1 expression (> 1%).

**Table 1:** Baseline information summary of included trials.

First author	Journal (year)	Region	Study period	Treatment	Disease	Phase	No. of patients	Tumor response	PD-L1 expression request
Hsu C	J Clin Oncol (2017)	International-Collaborated (Taiwan mainly)	–	Pembrolizumab: 10 mg/kg every 2 weeks	Recurrent or metastatic NPC failed on prior standard treatment	Ib	27	PR: 7 (25.9%)	Positive (PD-L1 > 1%)
								SD: 14 (51.9%)	
								PD: 6 (22.2%)	
Fang W	Lancet Oncol (2018)	Mainland China	2016.3–2017.9	Camrelizumab: escalating dose 1 mg/kg、3 mg/kg、10 mg/kg、200 mg every 2 weeks	Recurrent or metastatic NPC failed on prior standard treatment	I	93 (91 for tumor reponse evaluation)	CR: 2 (2%)	No
								PR: 29 (32%)	
								SD: 23 (25%)	
								PD: 37 (41%)	
Fang W	Lancet Oncol (2018)	Mainland China	2017.4–2017.8	Camrelizumab plus GP: Camrelizumab 200 mg d1 + gemcitabine 1 g/m^2^ d1、d8 + Cisplatin 80 mg/m^2^ d1	Treatment-naïve recurrent or metastatic NPC	I	23 (22 for tumor response evaluation)	CR: 1 (5%)	No
								PR: 19 (86%)	
								SD: 2 (9%)	
								PD: 0 (0)	
Huang J	Clin Cancer Res (2018)	Mainland China	2016.5–2016.12	Camrelizumab: 200 mg、400 mg or 600 mg every 2 weeks	Advanced or metastatic ESCC failed on at least one systemic treatment	I	30	CR: 1 (3.3%)	No
								PR: 9 (30%)	
								SD: 7 (23.3%)	
								PD: 13 (43.3%)	
Ma BB	J Clin Oncol (2018)	International-Collaborated (Hong Kong mainly)	2015.10–2016.6	Nivolumab: 3 mg/kg every 2 weeks	Recurrent or metastatic NPC failed on prior standard treatment	II	45 (44 for tumor response evaluation)	CR: 1 (2.3%)	No
								PR: 8 (18.2%)	
								SD: 15 (34.1%)	
								PD: 18 (40.9%)	
								NA: 2 (4.5%)	
Mo HN	Br J Cancer (2018)	Mainland China	2016.4–2016.12	Camrelizumab: 60 mg、200 mg、400 mg every 2 weeks	Advanced solid tumors failed at least one systemic treatment	I	36	CR: 2 (5.6%)	No
								PR: 7 (19.4%)	
								SD: 5 (13.9%)	
								PD: 22 (61.1%)	
Nie J	J Clin Oncol (2019)	Mainland China	2017.1–2018.7	Camrelizumab: 200 mg every 3 weeks	Relapsed /refractory Classical Hodgkin lymphoma failed at least two lines of previous therapy	II	19	CR: 6 (32%)	No
								PR: 11 (58%)	
								SD/PD: 2 (10%)	
Nie J	J Clin Oncol (2019)	Mainland China	2017.1–2018.7	Camrelizumab plus decitabine: Camrelizumab 200 mg d8 + decitabine 10 mg/day d1–d5 every 3 weeks	Relapsed /refractory Classical Hodgkin lymphoma failed at least two lines of previous therapy	II	67	CR: 37 (55.2%)	No
								PR: 16 (23.9%)	
								SD/PD: 14 (20.9%)	
Sheng XN	J Clin Oncol (2019)	Mainland China	2017.4–2018.4	Toripalimab plus Axitinib: Toripalimab 1 mg/kg、3 mg/kg every 2 weeks + Axitinib 5 mg twice daily	Chemotherapy-naïve or treated advanced mucosal melanoma	Ib	33 (29 for tumor response evaluation)	CR/PR: 14 (48.3%)	No
								SD: 11 (37.9%)	
								PD: 4 (13.8%)	
Shi YK	Lancet Oncol (2019)	Mainland China	2017.4–2017.11	Sintilimab: 200 mg every 3 weeks	Relapsed /refractory Classical Hodgkin lymphoma after two or more lines of previous therapy	II	96 (92 for tumor response evaluation)	CR: 31 (34%)	No
								PR: 43 (47%)	
								SD: 16 (17%)	
								PD: 2 (2%)	
Song YQ	Clin Cancer Res (2019)	Mainland China	2017.1–2017.9	Camrelizumab: 200 mg every 2 weeks	Relapsed /refractory Classical Hodgkin lymphoma failed to achieve a response or progressed after ASCT or failed at least two lines of systemic therapy	II	75	CR: 28 (37.3%)	No
								PR: 31 (41.3%)	
								SD: 14 (18.7%)	
								PD: 2 (2.7%)	
Song YQ	Leukemia (2019)	Mainland China	2017.4–2017.11	Tislelizumab: 200 mg every 3 weeks	Relapsed /refractory Classical Hodgkin lymphoma failed to achieve a response or progressed after ASCT or failed at least two lines of systemic therapy	II	70	CR: 44 (62.9%)	No
								PR: 17 (24.3%)	
								SD/PD: 9 (12.8%)	
Tang BX	J Hematol Oncol (2019)	Mainland China	2016.3–2016.12	Toripalimab: 1 mg/kg、3 mg/kg、10 mg/kg every 2 weeks	Metastatic melanoma or urologic cancers who were refractory to standard systemic therapy	I	36	CR: 1 (2.8%)	No
								PR: 7 (19.4%)	
								SD: 10 (27.8%)	
								PD: 18 (50%)	
Wu YL	J Thorac Oncol (2019)	International-Collaborated (Mainland China mainly)	2015.12–2016.11	Nivolumab: 3 mg/kg every 2 weeks	Non-small cell lung cancer progressed during/after platinum-based doublet chemotherapy	III	337 (338 for tumor response evaluation)	CR: 1 (0.3%)	No
								PR: 55 (16.3%)	
								SD: 121 (35.8%)	
								PD: 128 (37.9%)	
								NA: 33 (9.8%)	
Wang F	Ann Oncol (2019)	Mainland China	2016.12–2017.9	Toripalimab: 3 mg/kg every 2 weeks	Advanced adenocarcinoma of the stomach or gastroesophageal junction failed at least one line of systemic therapy	Ib/II	58	CR: 0 (0)	No
								PR: 7 (12.1%)	
								SD: 16 (27.6%)	
								PD: 35 (60.3%)	
Wang F	Ann Oncol (2019)	Mainland China	2017.12–2018.8	Toripalimab plus XELOX: Toripalimab 360 mg d1 + Oxaliplatin 130 mg/m^2^ d1 + Capecitabine 1000 mg/m^2^ bid d1–d14 every 3 weeks	Treatment-naïve advanced adenocarcinoma of the stomach or gastroesophageal junction	Ib/II	18	CR: 1 (5.6%)	No
								PR: 11 (61.1%)	
								SD: 4 (22.2%)	
								PD: 2 (11.1%)	

*NPC* nasopharyngeal carcinoma, *ESCC* esophageal carcinoma, *ASCT* autologous stem cell transplantation, *PD-L1* programmed death ligand-1, *CR* complete remission, *PR* partial remission, *SD* stable disease, *PD* progression diseas, *NA* not assessed.

### Pooled TRAEs and irAEs in whole cohorts

**Figure 2:**
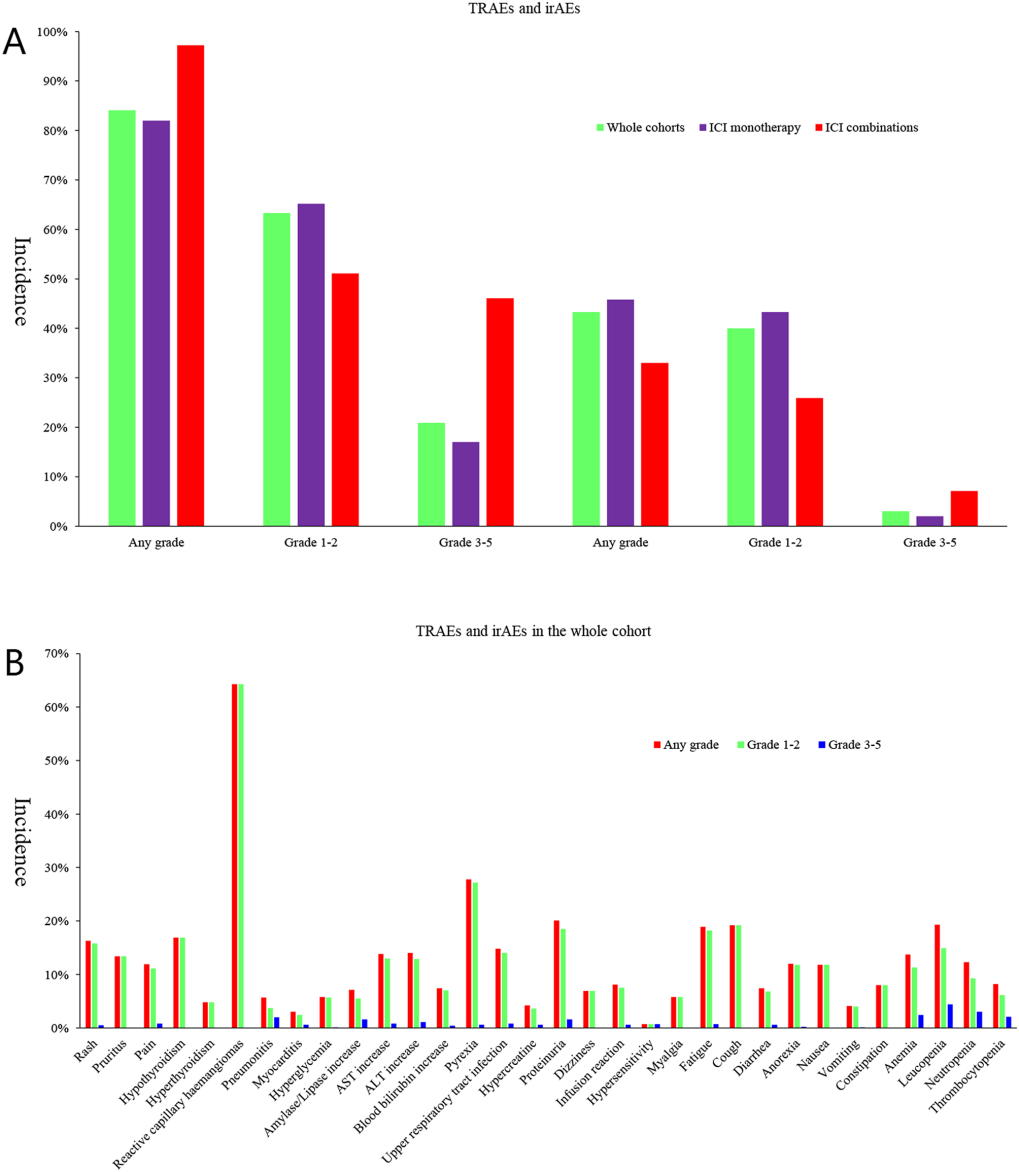
(**A**) Pooled incidence of treatment-related adverse events and immune-related adverse events among the 1063 patients; (**B**) Toxicity profiles in the 1063 patients.

We pooled all 13 studies together to evaluate any grade and grade 3–5 TRAEs and irAEs. Overall, 12 studies reported the data on any grade TRAEs (n = 1018) and grade 3–5 TRAEs were reported in all 13 studies (n = 1063). Any grade irAEs were shown in 7 studies (n = 424) and grade 1–2 irAEs in 8 studies (n = 451). Detailedly, the pooled incidence of any grade TRAEs, grade 1–2, grade 3–5 TRAEs, any grade irAEs, grade 1–2 irAEs and grade 3–5 irAEs were 84.1%, 63.3%, 20.9%, 43.3%, 40.0% and 3.0%, respectively (Fig. 2A). The highest incidence of any grade, grade 1–2 and grade 3–5 TRAEs were reactive capillary haemangiomas (64.3%), reactive capillary haemangiomas (64.3%) and leucopenia (4.4%), and the corresponding irAEs were reactive capillary haemangiomas (64.3%), reactive capillary haemangiomas (64.3%) and pneumonitis (2.0%) in whole cohorts (Fig. 2B, Supplementary Material, Table S1). In addition, 4.3% (44/1018) of patients experienced treatment discontinuation and only 8 (0.8%) patients experienced treatment-related death.

### Comparison of TRAEs and irAEs between ICI monotherapy and combination

**Figure 3:**
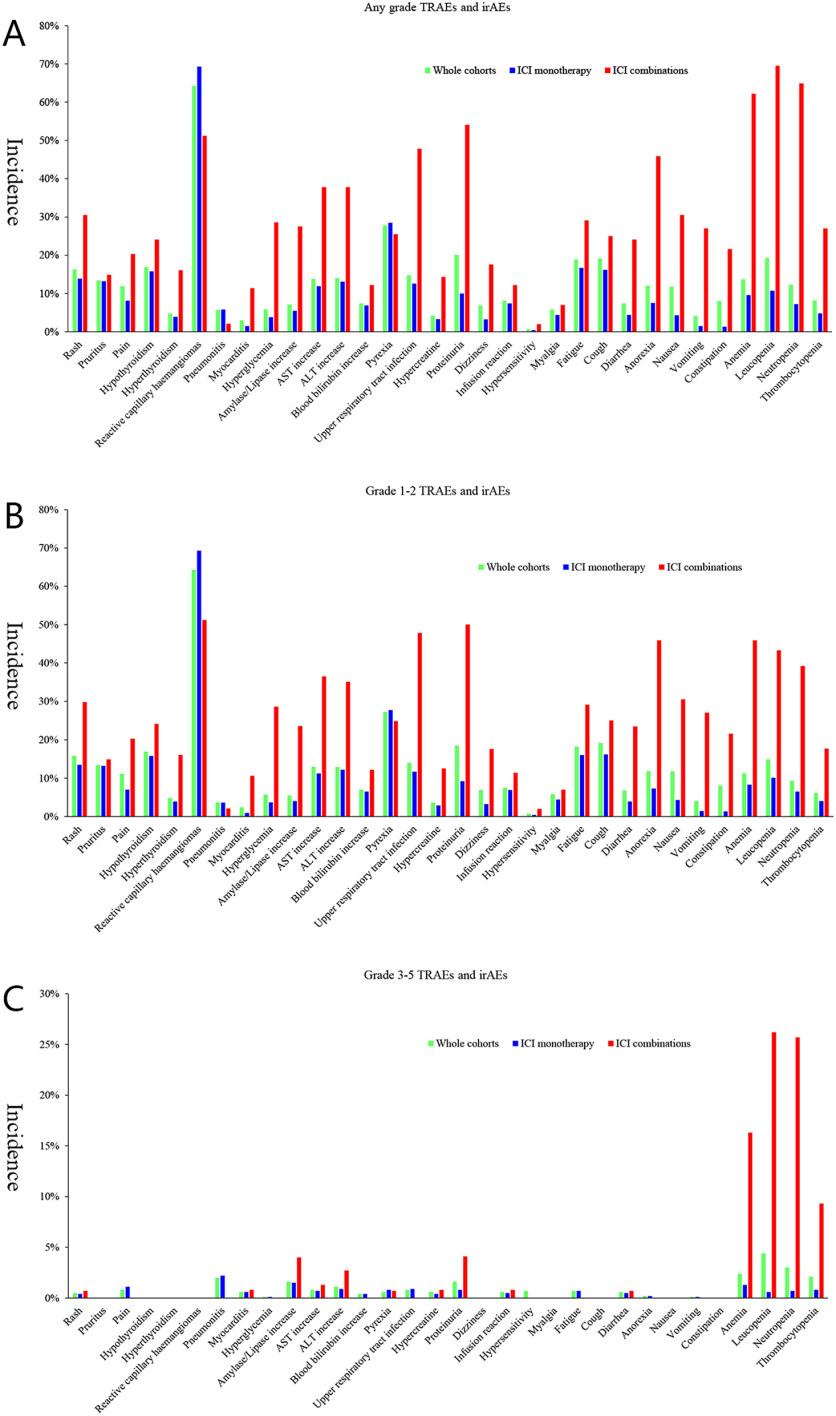
Treatment-related adverse events and immune-related adverse events between immune checkpoint inhibitor monotherapy and combination: (**A**) any grade; (**B**) grade 1–2; (**C**) grade 3–5.

The detailed profiles of TRAEs and irAEs between ICI monotherapy and combination were presented in Fig. 3. Overall, the any grade TRAEs (82.0% vs. 97.2%, *P* < 0.001), grade 3–5 TRAEs (17.0% vs. 46.1%, *P* < 0.001) and grade 3–5 irAEs (2.0% vs. 7.1%, *P* = 0.015) were significantly lower in ICI monotherapy group than those in ICI combination group, while grade 1–2 TRAEs (65.2% vs. 51.1%, *P* = 0.001), any grade irAEs (45.8% vs. 32.9%, *P* = 0.032) and grade 1–2 irAEs (43.3% vs. 25.9%, *P* = 0.003) were higher in ICI monotherapy group. Reactive capillary haemangiomas was the highest incidence of any grade AE for both whole cohorts (64.3%) and ICI monotherapy (69.3%, Fig. 3A) while ICI combinations have the highest incidence of hematological toxicities (Fig. 3A) such as anemia (62.2%), leucopenia (69.5%) and neutropenia (64.9%; Supplementary Material, Table S1). Generally, most TRAEs and irAEs were grade 1–2 except hematological AEs in ICI combinations group (Fig. 3B,C). ICI combinations significantly increased grade 1–2 rash (29.8% vs. 13.5%, *P* < 0.001), hypothyroidism (24.1% vs. 15.8%, *P* = 0.015), hyperthyroidism (16.1% vs. 3.9%, *P* < 0.001), myocarditis (10.6% vs. 0.9%, *P* < 0.001), hyperglycemia (28.6% vs. 3.7%, *P* < 0.001), amylase/lipase increase (23.5% vs. 4.0%, *P* < 0.001), Aspartate aminotransferase [AST] increase (36.5% vs. 11.2%, *P* < 0.001), Alanine aminotransferase [ALT] increase (35.1% vs. 12.2%, *P* < 0.001) and hypercreatine (12.5% vs. 2.9%, *P* < 0.001; Supplementary Material, Table S1). Besides, grade 3–5 irAEs except hematological toxicities were almost comparable between ICI monotherapy and combinations (all less than 5%, Fig. 3C).

### Comparison of TRAEs and irAEs between different ICIs

**Figure 4:**
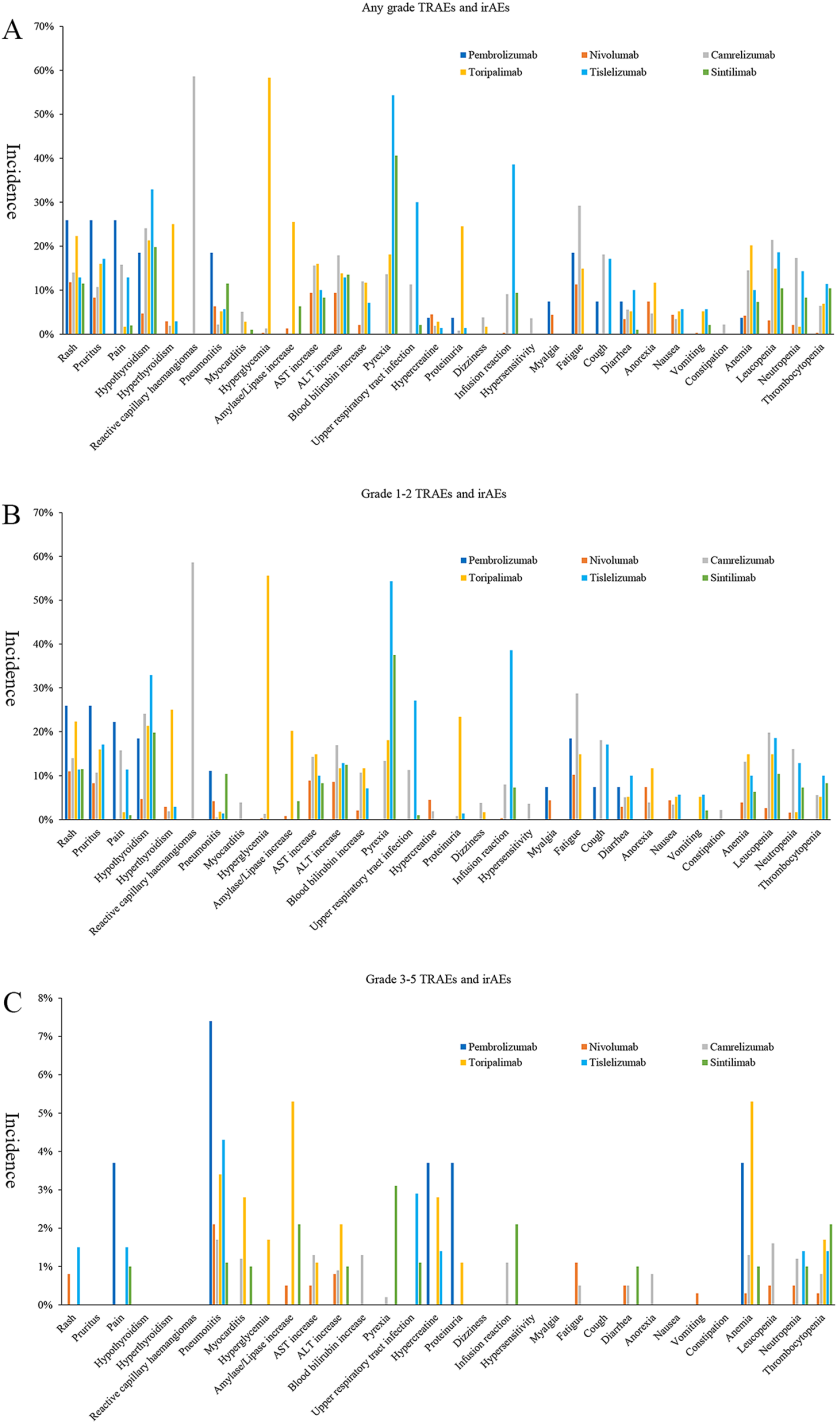
Treatment-related adverse events and immune-related adverse events between different immune checkpoint inhibitors: (**A**) any grade; (**B**) grade 1–2; (**C**) grade 3–5.

We further compared the TRAE and irAE profiles between different ICIs monotherapy (Fig. 4, Supplementary Material, Tables S2, S3). The overall incidence of TRAEs and irAEs of different ICIs were shown in. Generally, TRAEs and irAEs of different ICIs monotherapy were grade 1–2, and the incidence of grade 3–5 TRAEs and irAEs were almost below 5% (Fig. 4C). Intriguingly, the four domestic ICIs (camrelizumab, toripalimab, tislelizumab and sintilimab) had higher any grade (86.2–98.0% vs. 64.1–74.1%) and grade 1–2 TRAEs (58.5–79.8% vs. 44.4–53.7%) than abroad ICIs (pembrolizumab and nivolumab). Only camrelizumab reported the AE of grade 1–2 reactive capillary haemangiomas (58.6%), indicating that this AE should be camrelizumab-specific. Toripalimab has significantly higher incidences of grade 1–2 hyperglycemia (55.6%) and amylase/lipase increase (20.2%) than other ICIs, suggesting it may has higher impact on pancreas. Moreover, tislelizumab has the highest grade 1–2 infusion reaction (38.6%) and pyrexia (54.3%). For grade 3–5 irAEs, we should pay attention to pembrolizumab as it has the highest incidence of pneumonitis (7.4%). Also, grade 3–5 amylase/lipase increase (5.3%) and anemia (5.3%) caused by toripalimab should not be ignored. Besides, other grade 3–5 TRAEs and irAEs were very low.

## Discussion

To the best of our knowledge, our study is the first one to characterize the landscape of TRAEs and irAEs of ICI treatment in a predominantly Chinese cohort. Generally, ICI were well tolerated in Chinese patients and most TRAEs and irAEs were grade 1–2. As expected, ICI combined with chemotherapy or anti-angiogenesis could increase both TRAEs and irAEs. By comparing the toxicity profiles between different ICIs, nivolumab may be the safest as it achieves the lowest incidence of any grade TRAEs (64.1%) and grade 3–5 TRAEs (11.8%).

Different from the anti-tumor mechanism of cytotoxic or targeted drugs, ICI activated endogenous immune cells by blocking negative regulators to kill tumor cells. This unique mechanism should produce different toxicity profiles which is associated with immune. Reports from previous clinical trials indicated that ICI-related AEs were generally very mild and tolerable^15,33,34^. However, some toxicities such as myocarditis and pneumonitis were covert and have a very high fatality rate^19^. On the other hand, the durable response^18^ to ICIs means that patients would have longer exposure time, leading to a higher rate of ICI-related AEs. Therefore, it would be of great importance to provide an overview of ICI-related AEs for Chinese patients as such data are every rare. The incidences of any grade TRAEs of pembrolizumab and nivolumab were similar as that of previous study^35^, indicating the reliability of our study data. Possibly, the toxicity profiles from our study may help future selection of ICIs.

By indirectly comparing the toxicity data of pembrolizumab plus chemotherapy^10,12^ with pembrolizumab alone^15^, we could know that additional chemotherapy significantly increase grade 3–5 TRAEs (67.2–69.8% vs. 18%). In our study, however, we found that additional chemotherapy or anti-angiogenesis to ICI mainly increase grade 1–2 TRAEs and irAEs, which was inconsistent with previous findings. The main reason should be that only one study focused on the combination of platinum-based double regimen with ICI (n = 23)^21^ among the combination cohort, therefore leading to the underestimation of grade 3–5 TRAEs. Thus, we could not concluded that ICI combined with chemotherapy is safe in Chinese patients since our data is immature. We need more safety data from Chinese patients before we make a conclusion.

By comparing the toxicity profiles between different ICIs, we could find some specific AEs. For example, reactive capillary haemangiomas was only reported in camrelizumab-treated patients, indicating that it is camrelizumab-specific. Moreover, hyperglycemia, amylase/lipase increase and proteinuria were mainly in toripalimab-treated patients. Notably, pembrolizumab has the highest incidence of grade 3–5 pneumonitis and toripalimab has the highest incidence of grade 3–5 myocarditis. Thus, patients at high-risk of experience pneumonitis and myocarditis should avoid these two ICIs. Possibly, our mapping of ICI-related AEs would provide preliminary information for clinically individualized selection of ICIs. Besides drug itself, however, we should also pay attention the possibility that the differences of incidences of TRAEs and irAEs between ICIs were due to the difference of study design and sample size. Therefore, we need more data to drive mature conclusion.

Limitations of our study are also obvious. First, the number of study and sample are very small. Compared with the ongoing or finished studies abroad (n = 823, Fig. 1), the ICI-related trials in China are very lacking. Therefore, we should interpret the pooled incidence of ICIs-related TRAEs and irAEs prudently in case of insufficient statistical power. Second, our analysis was not based on the individualized patient data, therefore may underestimate or overestimate the incidence of some toxicities because many studies did not reported the AEs with an incidence below 5% or 10%. Large cohorts from real world may address this drawbacks. Moreover, due to the inclusion of different phases trials (phase I, II and III) and patients with different malignancies (solid and non-solid), the comparison results of different ICI monotherapy may be affected because the incidence and spectrum of irAEs of each ICI might vary significantly across studies and tumor types. Therefore, we should interpret the results of cross-ICIs comparison discreetly.

## Conclusions

We firstly described the landscape of ICI-related toxicity profiles in a predominantly Chinese cohort. Generally, ICI-related toxicities were mild, safe and tolerable. Each ICI has a different toxicity profile and we should focus on individualized selection. Moreover, we should be cautious about combination treatments of ICI with chemotherapy or anti-angiogenesis although our data showed this combination only increases grade 1–2 TRAEs and irAEs. Future studies with large sample were needed to provide more data and information.

## Supplementary information

Supplementary Legends

Supplementary Tables
