# Supplementary material for: Landscape of immune checkpoint inhibitor-related adverse events in Chinese population
Source: Sci Rep. 2020 Sep 23;10:15567. doi: 10.1038/s41598-020-72649-5 (PMC7511303; doi:10.1038/s41598-020-72649-5)
Supplement: Supplementary file 1 — Supplementary Legends [file 41598_2020_72649_MOESM1_ESM.docx]

**Supplementary material**

**Supplementary Table S1.** TRAEs and irAEs between ICI monotherapy and Combination. TRAE, treatment-related adverse event; irAE, immune-related adverse event; ICI, immune checkpoint inhibitor.

**Supplementary Table S2.** TRAEs and irAEs between different ICIs. TRAE, treatment-related adverse event; irAE, immune-related adverse event; ICI, immune checkpoint inhibitor.

**Supplementary Table S3.** Overall incidence of TRAEs and irAEs in different ICI monotherapies. TRAE, treatment-related adverse event; irAE, immune-related adverse event; ICI, immune checkpoint inhibitor.
