# Supplementary material for: Landscape of immune checkpoint inhibitor-related adverse events in Chinese population
Source: Sci Rep. 2020 Sep 23;10:15567. doi: 10.1038/s41598-020-72649-5 (PMC7511303; doi:10.1038/s41598-020-72649-5)
Supplement: Supplementary file 2 — Supplementary Tables [file 41598_2020_72649_MOESM2_ESM.docx]

**Landscape of Immune Checkpoint Inhibitor-related Adverse Events in Chinese Population**

**Running title: irAEs in Chinese Papulation**

**Authors and affiliations**

Li Li, M.D.,^1*^ Gang Li, M.D.,^2*^ Bin Rao, M.D.,^3^ An-Hui Dong, M.D.,^3^ Wei Liang, M.D.,^1^ Jin-Xian Zhu, M.D.,^1^ Mu-Ping Qin, M.D.,^2^ Wen-Wen Huang, M.D.,^3^ Jie-Ming Lu, M.D.,^3^ Zi-Fang Li, M.D.,^2^ Yao-Zhong Wu, M.D.^3^

1. Department of Radiation Oncology, Wuzhou Red Cross Hospital, 3-1 Xinxing First Road, Wuzhou 543001, People’s Republic of China.
2. Department of Medical Oncology, Wuzhou Red Cross Hospital, 3-1 Xinxing First Road, Wuzhou 543001, People’s Republic of China.
3. Department of Breast Surgery, Wuzhou Red Cross Hospital, 3-1 Xinxing First Road, Wuzhou 543001, People’s Republic of China.

**^*^Li Li and Gang Li Contributed equally to this study.**

**Corresponding author:**

Yao-Zhong Wu, Doctor

Department of Breast Surgery, Wuzhou Red Cross Hospital, 3-1 Xinxing First Road, Wuzhou 543001, People’s Republic of China.

**Telephone:** +86-0774-3832052

**E-mail**: [wuyaozhongwzhh@163.com](mailto:wuyaozhongwzhh@163.com)

Table S1. TRAEs and irAEs between ICI monotherapy and Combination.

| Toxicity | Whole cohort (%) | | | ICI monotherapy (%) | | | ICI combination (%) | | |
| --- | --- | --- | --- | --- | --- | --- | --- | --- | --- |
|  | Any grade | Grade 1-2 | Grade 3-5 | Any grade | Grade 1-2 | Grade 3-5 | Any grade | Grade 1-2 | Grade 3-5 |
| Overall incidence | 84.1 | 63.3 | 20.9 | 82.0 | 65.2 | 17.0 | 97.2 | 51.1 | 46.1 |
| Rash | 16.3 | 15.8 | 0.5 | 13.9 | 13.5 | 0.4 | 30.5 | 29.8 | 0.7 |
| Pruritus | 13.4 | 13.4 | 0 | 13.2 | 13.2 | 0 | 14.9 | 14.9 | 0 |
| Pain | 11.9 | 11.1 | 0.8 | 8.1 | 7.0 | 1.1 | 20.3 | 20.3 | 0 |
| Hypothyroidism | 16.9 | 16.9 | 0 | 15.8 | 15.8 | 0 | 24.1 | 24.1 | 0 |
| Hyperthyroidism | 4.8 | 4.8 | 0 | 3.9 | 3.9 | 0 | 16.1 | 16.1 | 0 |
| Reactive capillary haemangiomas | 64.3 | 64.3 | 0 | 69.3 | 69.3 | 0 | 51.2 | 51.2 | 0 |
| Pneumonitis | 5.7 | 3.7 | 0 | 5.8 | 3.6 | 2.2 | 2.1 | 2.1 | 0 |
| Myocarditis | 3.0 | 2.4 | 0.6 | 1.5 | 0.9 | 0.6 | 11.4 | 10.6 | 0.8 |
| Hyperglycemia | 5.8 | 5.7 | 0.1 | 3.8 | 3.7 | 0.1 | 28.6 | 28.6 | 0 |
| Amylase/Lipase increase | 7.1 | 5.5 | 1.6 | 5.5 | 4.0 | 1.5 | 27.5 | 23.5 | 4.0 |
| AST increase | 13.8 | 13.0 | 0.8 | 11.9 | 11.2 | 0.7 | 37.8 | 36.5 | 1.3 |
| ALT increase | 14.0 | 12.9 | 1.1 | 13.1 | 12.2 | 0.9 | 37.8 | 35.1 | 2.7 |
| Blood bilirubin increase | 7.4 | 7.0 | 0.4 | 6.9 | 6.5 | 0.4 | 12.2 | 12.2 | 0 |
| Pyrexia | 27.8 | 27.2 | 0.6 | 28.5 | 27.7 | 0.8 | 25.5 | 24.8 | 0.7 |
| Upper respiratory tract infection | 14.8 | 14.0 | 0.8 | 12.6 | 11.7 | 0.9 | 47.8 | 47.8 | 0 |
| Hypercreatine | 4.2 | 3.6 | 0.6 | 3.3 | 2.9 | 0.4 | 14.3 | 12.5 | 1.8 |
| Proteinuria | 20.1 | 18.5 | 1.6 | 10.0 | 9.2 | 0.8 | 54.1 | 50.0 | 4.1 |
| Dizziness | 6.9 | 6.9 | 0 | 3.2 | 3.2 | 0 | 17.6 | 17.6 | 0 |
| Infusion reaction | 8.1 | 7.5 | 0.6 | 7.4 | 6.9 | 0.5 | 12.2 | 11.4 | 0.8 |
| Hypersensitivity | 0.7 | 0.7 | 0 | 0.4 | 0.4 | 0 | 2.0 | 2.0 | 0 |
| Myalgia | 5.8 | 5.8 | 0 | 4.4 | 4.4 | 0 | 7.0 | 7.0 | 0 |
| Fatigue | 18.9 | 18.2 | 0.7 | 16.7 | 16.0 | 0.7 | 29.1 | 29.1 | 0 |
| Cough | 19.2 | 19.2 | 0 | 16.2 | 16.2 | 0 | 25.0 | 25.0 | 0 |
| Diarrhea | 7.4 | 6.8 | 0.6 | 4.4 | 3.9 | 0.5 | 24.1 | 23.4 | 0.7 |
| Anorexia | 12.0 | 11.8 | 0.2 | 7.5 | 7.3 | 0.2 | 45.9 | 45.9 | 0 |
| Nausea | 11.8 | 11.8 | 0 | 4.3 | 4.3 | 0 | 30.5 | 30.5 | 0 |
| Vomiting | 4.1 | 4.0 | 0.1 | 1.5 | 1.4 | 0.1 | 27.0 | 27.0 | 0 |
| Constipation | 8.0 | 8.0 | 0 | 1.3 | 1.3 | 0 | 21.6 | 21.6 | 0 |
| Anemia | 13.7 | 11.3 | 2.4 | 9.6 | 8.3 | 1.3 | 62.2 | 45.9 | 16.3 |
| Leucopenia | 19.3 | 14.9 | 4.4 | 10.7 | 10.1 | 0.6 | 69.5 | 43.3 | 26.2 |
| Neutropenia | 12.3 | 9.3 | 3.0 | 7.2 | 6.5 | 0.7 | 64.9 | 39.2 | 25.7 |
| Thrombocytopenia | 8.2 | 6.1 | 2.1 | 4.8 | 4.0 | 0.8 | 27.0 | 17.7 | 9.3 |

Abbreviations: TRAE, treatment-related adverse event; irAE, immune-related adverse event; ICI, immune checkpoint inhibitor.

Table S2. TRAEs and irAEs between different ICIs.

| Toxicity | Any grade (%) | | | | | | Grade 1-2 (%) | | | | | | Grade 3-5 (%) | | | | | |
| --- | --- | --- | --- | --- | --- | --- | --- | --- | --- | --- | --- | --- | --- | --- | --- | --- | --- | --- |
|  | Pembrolizumab | Nivolumab | Camrelizumab | Toripalimab | Tislelizumab | Sintilimab | Pembrolizumab | Nivolumab | Camrelizumab | Toripalimab | Tislelizumab | Sintilimab | Pembrolizumab | Nivolumab | Camrelizumab | Toripalimab | Tislelizumab | Sintilimab |
| Rash | 25.9 | 11.8 | 14.0 | 22.3 | 12.9 | 11.5 | 25.9 | 11.0 | 14.0 | 22.3 | 11.4 | 11.5 | 0 | 0.8 | 0 | 0 | 1.5 | 0 |
| Pruritus | 25.9 | 8.3 | 10.7 | 16.0 | 17.1 | NA | 25.9 | 8.3 | 10.7 | 16.0 | 17.1 | NA | 0 | 0 | 0 | 0 | 0 | NA |
| Pain | 25.9 | NA | 15.8 | 1.7 | 12.9 | 2.0 | 22.2 | NA | 15.8 | 1.7 | 11.4 | 1.0 | 3.7 | NA | 0 | 0 | 1.5 | 1.0 |
| Hypothyroidism | 18.5 | 4.7 | 24.1 | 21.3 | 32.9 | 19.8 | 18.5 | 4.7 | 24.1 | 21.3 | 32.9 | 19.8 | 0 | 0 | 0 | 0 | 0 | 0 |
| Hyperthyroidism | NA | 2.9 | 1.9 | 25.0 | 2.9 | NA | NA | 2.9 | 1.9 | 25.0 | 2.9 | NA | NA | 0 | 0 | 0 | 0 | NA |
| Reactive capillary haemangiomas | NA | NA | 58.6 | NA | 0 | NA | NA | NA | 58.6 | NA | 0 | NA | NA | NA | 0 | NA | 0 | NA |
| Pneumonitis | 18.5 | 6.3 | 2.2 | 5.2 | 5.7 | 11.5 | 11.1 | 4.2 | 0.5 | 1.8 | 1.4 | 10.4 | 7.4 | 2.1 | 1.7 | 3.4 | 4.3 | 1.1 |
| Myocarditis | 0 | 0 | 5.1 | 2.8 | 0 | 1.0 | 0 | 0 | 3.9 | 0 | 0 | 0 | 0 | 0 | 1.2 | 2.8 | 0 | 1.0 |
| Hyperglycemia | NA | 0.3 | 1.3 | 58.3 | NA | 0 | NA | 0.3 | 1.3 | 55.6 | NA | 0 | NA | 0 | 0 | 1.7 | NA | 0 |
| Amylase/Lipase increase | NA | 1.3 | NA | 25.5 | NA | 6.3 | NA | 0.8 | NA | 20.2 | NA | 4.2 | NA | 0.5 | NA | 5.3 | NA | 2.1 |
| AST increase | NA | 9.4 | 15.6 | 16.0 | 10.0 | 8.3 | NA | 8.9 | 14.3 | 14.9 | 10.0 | 8.3 | NA | 0.5 | 1.3 | 1.1 | 0 | 0 |
| ALT increase | NA | 9.4 | 17.9 | 13.8 | 12.9 | 13.5 | NA | 8.6 | 17.0 | 11.7 | 12.9 | 12.5 | NA | 0.8 | 0.9 | 2.1 | 0 | 1.0 |
| Blood bilirubin increase | NA | 2.1 | 12.0 | 11.7 | 7.1 | NA | NA | 2.1 | 10.7 | 11.7 | 7.1 | NA | NA | 0 | 1.3 | 0 | 0 | NA |
| Pyrexia | NA | NA | 13.6 | 18.1 | 54.3 | 40.6 | NA | NA | 13.4 | 18.1 | 54.3 | 37.5 | NA | NA | 0.2 | 0 | 0 | 3.1 |
| Upper respiratory tract infection | NA | NA | 11.3 | NA | 30.0 | 2.1 | NA | NA | 11.3 | NA | 27.1 | 1.0 | NA | NA | 0 | NA | 2.9 | 1.1 |
| Hypercreatine | 3.7 | 4.5 | 1.9 | 2.8 | 1.4 | NA | 0 | 4.5 | 1.9 | 0 | 0 | NA | 3.7 | 0 | 0 | 2.8 | 1.4 | 0 |
| Proteinuria | 3.7 | NA | 0.8 | 24.5 | 1.4 | NA | 0 | NA | 0.8 | 23.4 | 1.4 | NA | 3.7 | NA | 0 | 1.1 | 0 | NA |
| Dizziness | NA | NA | 3.8 | 1.7 | NA | NA | NA | NA | 3.8 | 1.7 | NA | NA | NA | NA | 0 | 0 | NA | NA |
| Infusion reaction | NA | 0.3 | 9.1 | 0 | 38.6 | 9.4 | NA | 0.3 | 8.0 | 0 | 38.6 | 7.3 | NA | 0 | 1.1 | 0 | 0 | 2.1 |
| Hypersensitivity | NA | 0 | 3.6 | NA | 0 | NA | NA | 0 | 3.6 | NA | 0 | NA | NA | 0 | 0 | NA | 0 | NA |
| Myalgia | 7.4 | 4.4 | 0 | NA | NA | NA | 7.4 | 4.4 | 0 | NA | NA | NA | 0 | 0 | 0 | NA | NA | NA |
| Fatigue | 18.5 | 11.3 | 29.2 | 14.9 | NA | NA | 18.5 | 10.2 | 28.7 | 14.9 | NA | NA | 0 | 1.1 | 0.5 | 0 | NA | NA |
| Cough | 7.4 | NA | 18.1 | NA | 17.1 | NA | 7.4 | NA | 18.1 | NA | 17.1 | NA | 0 | NA | 0 | NA | 0 | NA |
| Diarrhea | 7.4 | 3.4 | 5.6 | 5.2 | 10.0 | 1.0 | 7.4 | 2.9 | 5.1 | 5.2 | 10.0 | 0 | 0 | 0.5 | 0.5 | 0 | 0 | 1.0 |
| Anorexia | NA | 7.4 | 4.7 | 11.7 | NA | NA | NA | NA | 7.4 | 3.9 | 11.7 | NA | NA | 0 | 0.8 | 0 | NA | NA |
| Nausea | NA | 4.4 | 3.4 | 5.2 | 5.7 | NA | NA | 4.4 | 3.4 | 5.2 | 5.7 | NA | NA | 0 | 0 | 0 | 0 | NA |
| Vomiting | NA | 0.3 | 0 | 5.2 | 5.7 | 2.1 | NA | 0 | 0 | 5.2 | 5.7 | 2.1 | NA | 0.3 | 0 | 0 | 0 | 0 |
| Constipation | NA | NA | 2.2 | 0 | NA | NA | NA | NA | 2.2 | 0 | NA | NA | NA | NA | 0 | 0 | NA | NA |
| Anemia | 3.7 | 4.2 | 14.5 | 20.2 | 10.0 | 7.3 | 0 | 3.9 | 13.2 | 14.9 | 10.0 | 6.3 | 3.7 | 0.3 | 1.3 | 5.3 | 0 | 1.0 |
| Leucopenia | NA | 3.1 | 21.4 | 14.9 | 18.6 | 10.4 | NA | 2.6 | 19.8 | 14.9 | 18.6 | 10.4 | NA | 0.5 | 1.6 | 0 | 0 | 0 |
| Neutropenia | NA | 2.1 | 17.3 | 1.7 | 14.3 | 8.3 | NA | 1.6 | 16.1 | 1.7 | 12.9 | 7.3 | NA | 0.5 | 1.2 | 0 | 1.4 | 1.0 |
| Thrombocytopenia | NA | 0.3 | 6.4 | 6.9 | 11.4 | 10.4 | NA | 0 | 5.6 | 5.2 | 10.0 | 8.3 | NA | 0.3 | 0.8 | 1.7 | 1.4 | 2.1 |

Abbreviations: TRAE, treatment-related adverse event; irAE, immune-related adverse event; ICI, immune checkpoint inhibitor; NA, not available.

Table S3. Overall incidence of TRAEs and irAEs in different ICI monotherapies.

| ICI monotherapy | TRAEs (%) | | | irAEs (%) | | |
| --- | --- | --- | --- | --- | --- | --- |
|  | Any grade | Grade 1-2 | Grade 3-5 | Any grade | Grade 1-2 | Grade 3-5 |
| Pembrolizumab | 74.1 | 44.4 | 29.7 | NA | 37.0 | NA |
| Nivolumab | 64.1 | 53.7 | 11.8 | NA | NA | NA |
| Camrelizumab | 98.0 | 79.8 | 18.2 | 71.8 | 69.4 | 2.4 |
| Toripalimab | 86.2 | 58.5 | 27.7 | 19.1 | 17.0 | 2.1 |
| Tislelizumab | 92.9 | 71.4 | 21.5 | 38.6 | 38.6 | 0 |
| Sintilimab | 92.7 | 75.0 | 17.7 | 54.2 | 51.0 | 3.2 |

Abbreviations: TRAE, treatment-related adverse event; irAE, immune-related adverse event; ICI, immune checkpoint inhibitor; NA, not available.
